# Exploring experiences of binge-watching and perceived addictiveness among binge-watchers: a qualitative study

**DOI:** 10.1186/s12889-022-14789-z

**Published:** 2022-12-06

**Authors:** Yen-Jung Chang, Ching-Yi Peng

**Affiliations:** 1 Department of Health Promotion and Health Education, National Taiwan Normal University, 162, Section 1, Heping E. Rd., Taipei City, 106 Taiwan; 2 Department of Gerontological Health Care, National Taipei University of Nursing and Health Sciences, 365, Mingde Rd, Beitou District, Taipei City, 112 Taiwan

**Keywords:** Binge-watching, Addictive behaviors, Focus-group interview

## Abstract

**Background:**

Recent advances in technology and the Internet have led to the emergence of a phenomenon known as binge-watching. This qualitative study aims to explore experiences and perceptions of binge-watching behavior. The criteria of behavioral addiction were used to examine the characteristics of binge-watching behavior.

**Methods:**

We recruited 25 self-identified binge-watchers in Taiwan and conducted seven focus-group interviews with them in 2019 and 2020. Before their interview, the participants were asked to complete a brief questionnaire to collect information on their sociodemographic characteristics and binge-watching frequency.

**Results:**

The participants defined binge-watching behavior as consecutively watching episodes of shows with continuous content, rather than based on the time spent watching or the number of episodes watched. While they felt it may affect their daily routine, they mentioned almost no impacts on their health. Most participants emphasized the pleasure and social functions of binge-watching. This differs from previous studies, which have suggested an association between binge-watching and negative emotions. Notably, while most participants considered binge-watching to be an addictive behavior, they denied that they themselves were addicted.

**Conclusions:**

Our participants generally reported positive attitudes toward binge-watching. The addictiveness of binge-watching remains controversial. Further studies exploring the possibility of addictive binge-watching and potential mechanisms are warranted.

## Background

Watching television is considered to be one of the most common sedentary recreational activities among adults [1]. With technological advances that have popularized Internet services and mobile devices, the way that people watch television has changed, and temporal and spatial broadcast restrictions have lifted. Viewers are shifting from traditional cable television to online streaming platforms, leading to the emergence of online streaming giants like Netflix and Amazon. Using these platforms, viewers are able to escape the restrictions of scheduled programming and be in full control of what they watch and when they watch it. These changes have led to a new phenomenon called binge-watching [2].

As online streaming matures and binge-watching becomes increasingly common, consumers are demanding that providers release entire seasons at once to allow for faster watching. A survey reported that 50% of adult viewers aged 45 or younger could binge-watch an entire season of their favorite series in a single sitting [3]. The popularity of uninterrupted watching has been revealed in commercial survey studies. For example, 70% of viewers in the US binge-watch five episodes, and 80% of millennials binge-watch six episodes at a time [4]. Netflix users, on average, finish a season of their favorite shows in less than a week [5].

The Oxford Dictionary defines binge-watching as the practice of watching several episodes of a TV show on one occasion, usually on DVD or a digital streaming platform. However, the conceptualization and definition of binge-watching varies in the literature and consensus is yet to be achieved [2, 6–8]. Some scholars have used viewing time and number of episodes as measures of binge-watching, such as watching the same show consecutively for 2 h or more [9–11], or the uninterrupted viewing of several episodes of a show [12]. Others have proposed that a more comprehensive definition of binge-watching should take into account viewing time, equipment, and content [13]. Viewers’ perceptions of their binge-watching experiences have also been discussed, and it has been suggested that the conceptual view of binge-watching should be expanded to reflect more diverse viewing habits and experiences [14]. Despite the existence of this discussion, the experience and perceptions of binge-watching in the context of Taiwan remain underexplored, and this is the first research gap addressed by this study.

Binge-watching has become increasingly common in recent years, and its potential negative consequences (e.g., mental health problems) have raised public health concerns [15]. However, whether binge-watching is a behavioral addiction problem is another controversial topic in the existing literature. The Diagnostic and Statistical Manual of Mental Disorders Fifth Edition (DSM-V) characterizes behavioral addictions as excessive and repetitive behaviors, and the diagnostic standards for addictive behaviors proposed by Goodman and Brown [16] are widely referenced and discussed. Scholars stated that binge-watching is similar to substance addiction in that binge-watchers can build up a tolerance and are compelled to watch ever more episodes to be satisfied [11]. Other researchers have proposed concepts and measurement tools for problematic or addictive binge-watching based on the behavioral manifestations of addiction [17, 18]. However, it has been also argued that binge-watching should not be characterized as a problematic or addictive behavior [19, 20]. Given these unresolved debates, research should try to examine the applicability of the behavioral addiction notion to the conceptualization of binge-watching.

Before conducting further quantitative surveys to predict the health risks of binge-watching, we propose that qualitative studies like this one are necessary to elucidate how binge-watchers perceive this behavior in their own living contexts. A qualitative understanding of the manifestations and perceptions of binge-watching is essential because it will serve as the foundation of subsequent discussions about its adverse health effects or potentially addictive nature [21]. In this study, using a qualitative research design, we aimed to understand binge-watchers’ experiences and perceptions of binge-watching and explore the feasibility of examining binge-watching through the lens of behavioral addiction.

## Methods

### Data collection

The research team conducted a series of seven focus-group interviews to discuss the participants’ experiences of binge-watching behavior from July 2019 to January 2020. The focus groups were set up so that all participants in a group were within a limited age range. All interviews were hosted by the same researcher, who was accompanied by a transcriber. This study protocol was approved by the research ethics committee of the National Taiwan Normal University (#201905HS027). The study participants were informed about the research objectives and procedures before enrollment and notified of their interview time. Before their interview, the participants were asked to read and sign an informed consent form. They were also informed of their rights, which included that they were free to withdraw from the interview or ask the interviewer to stop the recording at any time. The interviews were held in a conference room or classroom on a university campus. Each interview lasted approximately 90 min, and all interviews were recorded in their entirety.

### Research tools

The interviews were semi-structured and were conducted using open-ended questions. The questions centered on the participants’ personal binge-watching experience, their perceived concept of binge-watching, the effects of binge-watching on daily life and health, their attitude towards binge-watching, and their self-identified susceptibility to addictive binge-watching. Before each focus-group interview, the participants were asked to complete a brief questionnaire to collect information on their sociodemographic characteristics and binge-watching frequency.

### Participants

We recruited participants through convenience sampling by distributing advertisements for adults in Taiwan who identified themselves as binge-watchers on various social networking platforms. People with educational training related to addiction science were excluded. Four of the six groups of participants were in the age range 20–29 years, so we established one additional focus group targeting participants aged ≥30 years to ensure that we did not miss any new information associated with middle-aged people specifically. We achieved data saturation after we had interviewed these seven focus groups, which involved 25 participants in total (32% men and 68% women; Table 1).

**Table 1:** Participant demographics

Code	Sex	Age	Occupation	Self-reported binge-watching frequency in the past month		
				Days per week	Hours per day (weekdays)	Hours per day (weekends)
1-A	F	28	Researcher	4	1.5	4
1-B	F	29	Researcher (part-time)	2	3	2
1-C	F	27	Korean language instructor; tour guide	7	*	*
2-A	F	20	University student	3	2	2
2-B	F	29	High school teacher	7	1	3
2-C	M	25	Research assistant	4	2	3
2-D	F	28	Employment officer	3	2	4
3-A	F	24	Unemployed	2–4	1–2	0
3-B	M	20	University student	3	2	4
3-C	F	20	University student	4	2–3	4–5
3-D	M	24	Employed (unspecified)	7	3	1
4-A	F	42	Social worker	4–5	1–3	1–4
4-B	F	30	Pharmaceutical researcher/developer	3	1	2
4-C	F	32	Insurance salesman	5	4	6
4-D	F	44	Hospital assistant	2	1	3
4-E	F	32	Full-time waiter	4	3	3
5-A	M	20	University student	1	1	1
5-B	M	24	Researcher (part-time)	5	2	5
5-C	M	24	Civil servant	6	1	4
6-A	M	36	Salesman	3	2	3
6-B	F	39	Researcher (part-time)	2	2	2
7-A	M	30	Insurance salesman	1–2	1	6
7-B	F	38	Civil servant	4	1	2
7-C	F	32	Part-time waiter	7	4	4
7-D	F	47	Homemaker (with a part-time job)	5	2	0

*No data provided by the participant

### Data consolidation and analysis

After each interview, the entire recording was transcribed. The lead researcher analyzed the transcripts, extracted key themes, and categorized and coded the data. A second researcher then reviewed the transcripts and assessed the suitability of each category and code, discussing differences in opinion with the first researcher until consensus was reached. Four major themes were identified: definition and experience, impact on daily life, behavioral addiction characteristics, and views on addictive binge-watching. For the addiction themes, data were categorized and analyzed based on the subjective views of the participants and the judgment criteria for addictive behaviors.

## Results

Among the 25 self-identified binge-watchers we interviewed, the average frequency of binge-watching in the month prior to the interview was 4.0 days per week, and the average time spent binge-watching was 1.9 hours per day during the week and 3.0 hours per day on the weekends. Apart from participant 7-D, whose preferred medium was cable television, the participants used streaming services on mobile devices (smartphones, tablets, and laptops). The findings of the interview data analysis are presented below according to the four major themes.

### Participants’ definition and experience of binge-watching

Three characteristics associated with binge-watching behavior were highlighted in the descriptions provided by the participants.

#### Continuity and immersion

All participants expressed the idea that binge-watching is a continuous and immersive behavior. We divided them into three categories based on their descriptions of such behavior. The first category comprised participants who maintained that watching content continuously and spending abundant time on watching constitutes binge-watching, regardless of the genre watched. The second category comprised participants who believed that binge-watching involves watching a show and later searching for information concerning that show; for example, the watcher might review cast information or research relevant merchandise. The third category comprised participants who described binge-watching as an uncontrollable behavior that negatively impacts viewers’ lives and causes them to neglect their responsibilities.

*I consider binge-watching to be a behavior that is difficult to stop. I think that continuously watching something for four hours or more constitutes the inability to stop. This behavior could negatively impact the viewer’s regular routine.* (Participant 6-B)

#### Content of shows

In studies conducted in other countries, binge-watching refers to the continuous, uninterrupted viewing of fictional series. However, the participants of this Taiwanese study had a broader interpretation of the term. In addition to mentioning the features of uninterrupted viewing and planned viewing, the participants also emphasized the viewing of continuous content. When asked “What have you been binge-watching recently?”, their responses included variety shows, reality shows, and anime. This suggested that their concept of binge-watching was broader than simply watching fictional series. Nineteen of the participants asserted that only watching fictional series constituted binge-watching, while others said that planned viewing, regardless of content, constituted binge-watching, and genre did not affect their perceptions of binge-watching.

Several of the participants said that only watching programs with continuous content constituted binge-watching, and that watching programs with discrete content, such as the news or variety shows, did not constitute binge-watching. Participant 7-A noted that “Watching ‘Perfect Neighbors’, [a Taiwanese soap opera], constitutes binge-watching, but watching NBA games does not. I would also consider watching ‘One Piece’, [a Japanese anime television series], binge-watching, but not watching a movie on HBO at a certain time.” Dramas and anime series present overarching storylines that span multiple episodes, while NBA games are not interconnected and random movies on HBO are independent (discrete) content.

#### Planned behavior

The researcher asked the participants “In your opinion, what is the difference between watching TV and binge-watching?” to understand their perspectives on binge-watching. The participants said that instead of passively watching TV, viewers are more in control when they binge-watch. It is therefore a planned behavior or a conscious decision. Binge-watchers also prefer to binge-watch on mobile devices.

*I am in control when I binge-watch. You can choose to binge-watch at six in the morning or six in the evening. By comparison, we have to follow set broadcast schedules when we watch TV. To me, there’s a big difference.* (Participant 4-A)

### Impacts of binge-watching on daily life

Based on the interview results, we classified the impact of binge-watching on the viewers’ life into three categories.

#### Daily routine

Most of the participants agreed that binge-watching affected their daily routines. The most common periods to binge-watch were after school or work and before bed. The participants also said that binge-watching late into the night or after their usual bedtime negatively affected their mood the following day. However, most of them did not think that binge-watching significantly impacted their health.

*I think binge-watching is physiologically taxing. For example, if you were to binge-watch late into the night, you’d be more likely to sleep until noon the next day. You’d then feel less sleepy at nine or ten in the evening and continue to binge-watch into the night, waking up again the next day at noon. Your daily routine is thus completely wrecked.* (Participant 2-A)

#### Stimulus and pleasure

A number of the participants said that binge-watching deeply influenced their way of thinking and values. Some stated that they were nourished and that their creativity was stimulated by the shows they watched, while others said they were more likely to feel empathetic because their shows revealed situations that could occur in real life.

*By watching shows, I’m able to gauge my own mental state in certain situations. For example, I would think about how I would feel if I had a friend or family member with a terminal illness and the doctor told me that it could not be treated. From another perspective, I think about how I would resolve this issue when it arises.* (Participant 7-C)

The participants also said that binge-watching is entertaining, and the pleasure associated with it prompts them to engage in other activities. A number of them stated that their binge-watching had prompted them to buy merchandise or travel to filming locations. Others said that binge-watching had motivated them to learn a foreign language, explore different cultural backgrounds, acquire professional knowledge in fields such as law and medicine, and understand social trends.

#### Social interaction

When discussing the effects of binge-watching, almost all the participants agreed that binge-watching had improved their interpersonal relationships because it gave them common interests to talk about with their friends and colleagues.

*We developed similar interests, so we had more to talk about. A good show can truly improve your relationships with your friends. There is a stark difference between the relationships with friends that I share a show with and those that I don’t.* (Participant 6-A)

### Characteristics of binge-watching and addictive behaviors

We referenced the behavioral addiction characteristics proposed by Griffiths [22, 23] and Goodman [16] to analyze the participants’ binge-watching experiences. We consolidated five characteristics: mood modification, tolerance, withdrawal, loss of control, and conflict (Table 2).

**Table 2:** Behavioral addiction concepts applied in analyzing binge-watching behavior characteristics

Concept	Description
Mood modification ^a,b^	Sense of pleasure derived from engaging in the behavior
Tolerance ^a,b^	Need to increase the intensity or frequency of the behavior to feel satisfied
Withdrawal ^a,b^	Negative reactions, such as irritation or restlessness, to not engaging in the behavior
Loss of control ^a^	Loss of control when engaging in the behavior
Conflict ^b^	Conflict with oneself or others about engaging in the behavior

^a^Proposed by Goodman

^b^Proposed by Griffiths

#### Mood modification

The participants described feeling excitement, a sense of urgency, and an inability to stop when they were binge-watching. These emotions are consistent with the behavioral addiction characteristic of mood modification proposed by Goodman and Griffiths.

*When I finish an episode, I feel a strong urge to watch the next episode. This feeling is less prevalent for shows I randomly pick to watch casually. The latter instance does not constitute binge-watching.* (Participant 1-C)

#### Tolerance

We found that the participants did need to increase their binge-watching intensity or duration over time to find satisfaction. However, the amount of time they spent binge-watching may have depended on whether they were employed. Those who reported a gradual increase in binge-watching duration were largely students. The binge-watching behaviors of married or employed participants over the age of 30 were restricted by their jobs and families and they were less likely to allocate increasing amounts of time to binge-watching.

*I spend a lot of time binge-watching. Binge-watching is extremely attractive. I find myself searching for other shows featuring my favorite actors, resulting in spending even more time binge-watching.* (Participant 2-B)

*I have a busy job and a family with kids, so I have responsibilities that I cannot get out of. I really want to carry on, but life forces me to stop.* (Participant 6-B)

#### Withdrawal

Several of the participants said they developed negative emotions when they were unable to binge-watch. They explained that they felt bored, sad, and disappointed when they finished a show, and some asserted that these negative emotions provided motivation for them to search for new shows. When they were asked how they would feel if they were prohibited from binge-watching, several responded that life would be dull. When asked how they would feel if they were forced to stop watching mid-episode, a number of them said they would have strong negative emotions such as anger, helplessness, and regret. Two participants (1-B and 2-A) said they would turn to searching for relevant articles and reviews if they were forced to stop binge-watching, to alleviate some of their negative emotions.

*I would feel annoyed if my mom called me for no reason while I was binge-watching.* (Participant 6-B)

*I feel sad when I finish a show. K-drama forums mention that the best way to recover from this sadness is to find something else to watch.* (Participant 2-B)

#### Loss of control

Several of the participants subjectively defined binge-watching as an uncontrollable behavior that has a negative impact on daily life and causes people to neglect their responsibilities. Their definitions were consistent with Goodman’s (1990) concept of behavioral addiction: a feeling of lack of control while engaging in a behavior. However, when they described their own binge-watching situations, most of the participants said their current binge-watching habit was controllable. They generally described their past binge-watching habits or the habits of others as uncontrollable, however.

*I suspect that my girlfriend is addicted. She would binge-watch for three days straight. She’d stay up late or binge-watch for seven hours at a time. I’d say she’s addicted.* (Participant 5-C)

*I was possibly addicted when I was young. Back then, I’d watch 10–12 episodes of a Japanese drama at a time. Each episode was an hour back then. I would finish an entire season in a single day.* (Participant 6-A)

#### Conflict

Conflict related to binge-watching is primarily between binge-watchers and their families, and less frequently between binge-watchers and their colleagues. According to the interview results, family members who were not binge-watchers themselves generally regarded binge-watching negatively and felt that it was not a good way to spend one’s time, often criticizing the behavior or prohibiting others from doing it. In contrast, family members who were binge-watchers themselves shared their views on the shows and storylines with the participants. Several of the participants also said they felt conflicted in themselves about their binge-watching, or that they felt guilty about not being able to exercise self-control.

*They would ask why I was binge-watching instead of sleeping. They’d question what good the show could do for my life.* (Participant 4-E)

*In reality, I feel pretty useless for not doing something productive. I’d binge-watch close to exams and criticize myself for binge-watching instead of studying.* (Participant 4-C)

### Views on addictive binge-watching

When the participants were asked if they thought that binge-watching was an addictive behavior, 19 responded in the affirmative. When they were asked if they had a binge-watching addiction themselves, most replied that they may have been addicted in the past but had kicked the habit now. The participants agreed that binge-watching is an addictive behavior because of the negative emotional impact of an inability to binge-watch, the inability to control the duration of binge-watching, the constant thinking about the current show, the eagerness to watch the next episode, the impact on daily life, and the resulting neglect of responsibilities. We noticed that some of the participants referred to others’ binge-watching experiences when discussing it as an addiction but drew on their own experiences when discussing it as a self-constrained or planned behavior. Respondent 1-B asserted, “The binge-watching is an addiction is when you are unable to wake up in the morning because you watched 10 episodes the night before.” However, he did not feel that his own binge-watching behavior was addictive, stating, “I finish all my work and go to sleep when I have to. My binge-watching behavior does not affect my life.” Participant 5-A, however, said repeatedly, “I always feel the need to watch the next episode. I think I may be addicted.” He also stated that his binge-watching behavior was planned, however, saying, “I choose one day of the week to binge-watch for half the day or the entire day. I watch three to four episodes at a time, and I generally take a break between each episode.”

## Discussion and conclusions

Research into emerging binge-watching behavior is developing, but conclusive definitions and measuring tools have yet to be agreed upon [6–8]. In this study, we conducted qualitative interviews to explore the characteristics of binge-watching behavior. It has been noted that most of the participants in other binge-watching studies have been single, female, and/or students [24, 25]. In this study, we deliberately recruited middle-aged and employed participants in addition to young students. The data revealed different binge-watching patterns among students and employed people. In the latter group, time spent binge-watching may have been influenced by work and family, while the students were unconstrained and thus more likely to increase their binge-watching intensity or duration to achieve satisfaction. Besides, we have assumed little effects of the COVID-19 pandemic on this study because it had not yet begun during the study period. All the interviews had been completed by January 2020, while the pandemic and nationwide lockdown regulations began only in April 2020 in Taiwan.

A recruitment issue that represents a limitation of this study is that we tried to arrange the focus groups such that the members of each group were relatively homogeneous, and we planned to recruit between four and six participants for each focus group. However, some groups comprised fewer than four participants because some people failed to arrive. This reduced number of interview participants may have affected the group’s discussions.

Our participants had broader and more relaxed definitions of binge-watching than studies conducted overseas, which have generally defined it based on duration [2, 26–29]. One of our research aims was to understand how the viewers themselves define what it means to binge-watch. Our participants perceived that they were binge-watchers based on their continuous, immersive, and planned viewing of the show with continuous content. When sharing their experiences, they disclosed the emotions of feeling excitement, a sense of urgency, and an inability to stop when they were binge-watching. Thoughts about the number of hours or episodes they watched was not highlighted in the focus group discussions. They also expressed the view that binge-watching can include various kinds of programs, not only fictional series, as long as the show content has continuity. These subjective views of binge-watching, which focus instead on the duration of viewing or the number of episodes, should be considered in future studies that attempt to measure binge-watching behavior.

Scholars have expressed concern that binge-watching can have a negative impact on health, but the participants of this study presented relatively positive perceptions of the behavior. Generally, they agreed that binge-watching affects daily routines, but not that it affects health. In fact, they felt that it has positive effects on emotional and social health, and their responses highlighted the idea that binge-watching is fun and improves social relations. A number of previous studies have concluded that binge-watching negatively affects mental health, stating that it leads to depression and provides an outlet for people with depression to escape reality [30–33]. Other studies have suggested that binge-watchers might tend to watch more episodes to alleviate loneliness [34, 35]. However, in this study, the participants seldom mentioned negative emotions when discussing their binge-watching experiences and motivations.

Our findings may contribute to the discussion about the potentially addictive nature of binge-watching. Comparing the interview responses with the information collected in the brief questionnaire administered prior to the interview, we found that the participants with a higher binge-watching frequency (5 days a week or more) or duration (3 h or more on both weekdays and weekends) were more likely to exhibit behavioral addiction characteristics. For example, participants 1-C and 4-C both said that binge-watching affected their lives more, in terms of their daily routine, sleep, work, and university participation. They also said it was difficult to control the time they spent binge-watching, and that binge-watching affected their mood considerably. While new episode releases positively affected their mood (participant 4-C), stopping binge-watching affected in negatively (participant 1-C).

Most of our participants believed that binge-watching could be an addictive behavior, but most of them did not believe that they were addicted themselves. Rather, they mentioned that they knew others with a binge-watching addiction. Similarly, several participants described binge-watching as an uncontrollable behavior with negative effects with reference to other viewers, while they described themselves as being in control and said they planned their binge-watching sessions so that they did not affect their jobs or daily schedules. These observations imply that the participants had contradictory perceptions of binge-watching and addiction. We recommend that further research is conducted to investigate whether these contradictions occur in viewers in other cultural contexts and whether they are related to negative attitudes or even stigma concerning binge-watching.

## Data Availability

The data presented in this study are available on reasonable request from the corresponding author.
